# OptiNeoCare: optimisation of routine care in the management of severe perinatal asphyxia in full-term or near-term newborns – study protocol for analysis of suboptimal care by confidential inquiries and e-self report

**DOI:** 10.1136/bmjopen-2025-106093

**Published:** 2025-07-05

**Authors:** Isabelle Guellec, Pierre-Yves Ancel, Hendy Abdoul, Charles Garabedian, Eric Verspyck, Cyril Huissoud, Marie Delnaud, Blanche Graesslin, Thomas Desplanches, Gilles Cambonie, Pierre Tourneux, Thierry Debillon, Ayoub Mitha, Gauthier Loron, Géraldine Favrais, Maliha Badr, Sophie Chapeliere, Marie Brasseur-Daudruy, Max Gonzalez Estevez, Agnes Rigouzzo, Pierre Delorme, Gilles Kayem

**Affiliations:** 1 Department of Medicine, Nice Côte d’Azur University, Nice, France; 2 Department of Neonatal Intensive Care, University Hospital Centre Nice, Nice, France; 3 OPPaLE (Obstetric, perinatal, paediatric life course epidemiology), INSERM UMR 1153, Paris, France; 4 URC Paris Descartes Necker/Cochin, Assistance Publique - Hopitaux de Paris, Paris, France; 5 Department of Obstetrics, ULR 2694-METRICS, CHU Lille, University of Lille, Lille, France; 6 Rouen Univ Hosp, Rouen, France; 7 Croix Rousse University Hospital, Lyon, France; 8 Department of Obstetrics and Gynaecology, Dijon University Hospital, Dijon, France; 9 Midwife, University of Reims, Reims, France; 10 CHU Dijon, Dijon, France; 11 Geneva School of Health Sciences, HES-SO University of Applied Sciences and Arts Western Switzerland, Geneva, Switzerland; 12 Neonatology and Neonatal Intensive Care Unit, Montpellier University Hospital Centre, Montpellier cedex 5, France; 13 Neonatal Intensive Care Unit, University Hospital Center of Amiens, Jules Verne University of Picardy, 80054, Amiens Cedex 1, Amiens, France; 14 Univ Grenoble Alpes, Grenoble, France; 15 Neonatal Intensive Care Unit, Tours University Hospital, Tours, France; 16 Neonatal Medicine and Pediatric Intensive Care, CHU de Reims, Reims, France; 17 Department of Neonatology, Caen University Hospital, Université de Caen Normandie, UFR Médecine, Caen, France; 18 Department of Neonatal Medicine and Pediatric Intensive Care, Arnaud de Villeneuve Hospital, Montpellier University Hospital, Montpellier, France; 19 Department of Pediatric Radiology, AP-HP, Bicêtre Hospital, Le Kremlin Bicêtre, France; 20 Department of Imaging, American Hospital of Paris, Neuilly, France; 21 Department of Pediatric-Radiology, Rouen University Hospital, Rouen, France; 22 Department of Anesthesia in Gynecology and Obstetrics, Lille University Hospital, Lille, France; 23 Department of Anesthesia in Gynecology and Obstetrics, Trousseau Hospital, Paris, France; 24 Sorbonne Université, AP-HP, Department of Gynecology and Obstetrics, Trousseau Hospital, Paris, France

**Keywords:** EPIDEMIOLOGY, Quality in health care, Neonatal intensive & critical care, OBSTETRICS, Clinical Protocols

## Abstract

**Abstract:**

**Introduction:**

Severe perinatal asphyxia at term or near term remains a critical public health issue, associated with high risks of neonatal death and hypoxic-ischaemic encephalopathy (HIE). Despite improved clinical guidelines, suboptimal care persists in many cases, and previous audits have demonstrated that up to 50% of asphyxia cases could be associated with suboptimal care. OptiNeoCare is a French study which aims to assess the prevalence and determinants of suboptimal obstetric and neonatal care and evaluate its potential impact on neonatal outcomes.

**Materials and methods:**

This prospective, population-based observational study will include newborns ≥36 weeks’ gestation with severe perinatal asphyxia across 12 French perinatal networks (213 maternity units). Inclusion criteria comprise neonatal death or moderate/severe HIE with confirmed biochemical markers of asphyxia. Data will be collected prospectively from labour wards, transport teams and neonatal intensive care units using an electronic case report form, and the in-situ team will be invited to complete a morbi-mortality review (MMR). Approximately 336 cases will be included over 12 months, with 25% randomly selected for confidential enquiry by two experts. The quality of care will be assessed based on a structured classification of medical errors (diagnostic, therapeutic, preventive and systemic) by a panel of experts including an obstetrician or midwife and a paediatrician. Root cause analysis will identify determinants of suboptimal care. A concordance analysis will compare findings from MMRs and confidential enquiries. Statistical analysis will include multivariable logistic regression to explore associations between care quality and neonatal outcomes.

**Ethics and dissemination:**

Ethical approval was granted by the Ethics Committee for Research in Obstetrics and Gynaecology. Informed non-opposition is required from participants. Results will be shared with participating centres, healthcare professionals and through scientific dissemination.

**Trial registration number:**

ClinicalTrials.gov ID: NCT06322732.

**STRENGTHS AND LIMITATIONS OF THIS STUDY**

- This study will focus on severe perinatal asphyxia, a critical yet rare condition with high morbidity and mortality, to evaluate the quality of care and identify opportunities for improving neonatal outcomes.
- This study will be a population-based multicentre study involving 213 maternity units across 7 French regions, ensuring comprehensive national coverage and generalisability of findings.
- Prospective and detailed data collection on obstetric, neonatal, transport and organisational factors will allow for indepth analysis of care pathways.
- The methods will associate an in-situ morbi-mortality review and a standardised expert review process using a confidential enquiry methodology to assess the quality of perinatal care.
- A perinatal study combining obstetric, neonatal and transport data collection through structured questionnaires.

## Introduction

 Perinatal asphyxia at term is a severe condition that can result in peripartum death or a birth with critical distress, leading to neurological impairment known as hypoxic-ischaemic encephalopathy (HIE), which occurs in approximately 1.6 per 1000 births.^1^ As an unexpected and urgent situation, obstetric and neonatal management plays a crucial role in determining neonatal and neurological outcomes.^2^ Despite significant improvements in prognosis over recent years,^3^ the condition remains severe, with an estimated mortality rate of 15–20% and a risk of moderate to severe disability in 30% of survivors.^4^

The management of severe perinatal asphyxia involves multiple risk factors that have been identified as contributors to suboptimal care in this particularly vulnerable patient population. Batlle *et al*’s clinical audit of obstetric practice in France in 2010, conducted through peer review, revealed that half of the audited cases of peripartum asphyxia were deemed ‘possibly or certainly avoidable’.^5^ Similar findings have been reported in studies from Denmark and Sweden.^6 7^ Additionally, Chevallier *et al*^8^ in France demonstrated that approximately 35% of neonates requiring therapeutic hypothermia did not receive it in accordance with national guidelines.^9^ The analysis of these suboptimal care practices aims to identify systemic factors leading to errors and to propose solutions to prevent similar cases in the future.^10^

Corrective actions have been implemented based on these studies. ‘Each Case of Asphyxia Could Be Used as a Learning Example’ is the title of an article proposing clinical practice recommendations derived from perinatal asphyxia case audits.^11^ In Queensland, the implementation of a national fetal heart rate monitoring education programme significantly reduced the incidence of HIE from 250 to 160 events per 100 000 live births.^12^ In the UK, a pragmatic approach has been initiated to improve outcomes in perinatal asphyxia. *Each Baby Counts* (https://www.rcog.org.uk/eachbabycounts) is a national platform that records all cases of perinatal asphyxia and HIE while collecting data on suboptimal care practices. This initiative aims to enhance care quality both locally and nationally.

These experiences suggest that the identification of suboptimal obstetric and neonatal care through voluntary institutional engagement can improve prevention and management. In France, the analysis of severe adverse events is conducted through morbidity and mortality reviews (MMR), which remain internal to healthcare institutions and have a limited national impact. However, the prevalence, causes and risk factors—whether individual or related to healthcare organisation—as well as the evaluation of suboptimal care at a population level, have not been systematically assessed. The confidential enquiry remains the gold standard for identifying suboptimal care. However, to date, no study has directly compared this method with routine in-situ MMRs in neonates.

The aim of this study is to describe cases of perinatal death or HIE, analyse associated risk factors and evaluate the quality of obstetric and neonatal care.

## Methods and analysis

OptiNeoCare is a prospective population-based observational study with an assessment of the optimality of the obstetric and neonatal care pathway.

### Data analysis plan

The first inclusions are expected to begin in May 2025. Four expert panel meetings will be held at regular intervals to assess the quality of care through confidential enquiries, conducted between the third quarter of 2025 and the third quarter of 2026. Data analysis will begin in the fourth quarter of 2026, with results expected to be disseminated by the end of 2027.

### Design

All included cases will be assessed through an in-situ review using a standardised online questionnaire, completed by the designated referent from each maternity unit. These digital records will be analysed, compiled and summarised by the *OptiNeoCare* steering committee. For cases undergoing a local MMR, detailed information regarding the optimality of care will be documented and analysed.

For cases randomised for the confidential enquiry, two external assessors (one paediatrician and one obstetrician or midwife) will conduct an on-site review. The results of the assessment will then be entered directly into Cleanweb for each mother-newborn pair. The identification of suboptimal care and the determination of case preventability will be discussed by an expert panel. This evaluation will be based on recognised criteria (clinical guidelines, consensus statements, best practice recommendations or expert opinion in the absence of literature).

### Setting

This is a population-based study conducted across seven regions of France, involving 12 perinatal networks and a total of 213 maternity units: 32 level-3, 108 level-2, 69 level-1 units and four midwifery-led birth centres. These facilities contributed to the inclusion of 336 patients. Among the 213 participating maternity units, 44 are located in the Île-de-France region (including the city of Paris) and 169 in other regions: Pays de la Loire, Auvergne-Rhône-Alpes, Hauts-de-France, Bourgogne, Occitanie and Normandie. Each unit is affiliated with its respective perinatal referral centre.

### Patient and public involvement

All parents will be informed about the study through public notices (posters displayed in maternity wards and neonatal units) and via an individual information leaflet provided at admission, indicating that a study on the quality of care surrounding perinatal asphyxia is currently being conducted in the maternity unit where they will deliver.

The aggregated results of the survey will be presented at medical conferences that include parent committees, such as the SOS Prema and Hospitalized Babies Association, which participates in the French Society of Neonatology congress and The European Foundation for the Care of Newborn Infants, which is involved in organising European congresses. This will facilitate communication of the results to stakeholders. Additionally, the findings will be shared with the general public through information networks such as mainstream newspapers and other media outlets.

### Study population

The study population will include term or near-term newborns (≥36 weeks of gestation) presumed to be alive at the onset of labour and who presented with severe perinatal asphyxia, defined by both of the following criteria:

1. Clinical severity of neonatal outcome. At least one of the following criteria had to be met:
  Neonatal death during the initial hospital stay (from birth until discharge or before day 28 of life, whichever came first) or moderate to severe HIE or hospitalisation within the first week of life due to HIE or seizures attributable to a peripartum event.
2. Biological evidence of perinatal asphyxia. At least one of the following:
  Arterial (or, if not available, venous) cord blood pH ≤7.10 or base deficit ≥16 mmol/L or lactate ≥11 mmol/L within the first 3 hours of life or in the absence of biochemical documentation of metabolic acidosis, one of the following criteria: Apgar score ≤5 at 5 min of life or need for neonatal resuscitation, defined as ventilatory support at birth and Apgar score ≤7 at 10 min. If no biochemical or Apgar data were available in the medical record (eg, in cases of home births): inclusion will be based on neonatal outcome (death, HIE or neonatal seizures).

Moderate or severe HIE is defined according to the criteria of the French Society of Neonatology, in the context of perinatal asphyxia, as follows: signs of cortical dysfunction: lethargy with diminished response to stimulation or coma with absent response; and at least one of the following: abnormal muscle tone, abnormal primitive reflexes, weak or absent sucking reflex, clinical seizures.

Peripartum event is defined as a sentinel event, such as uterine rupture, maternal haemorrhage or collapse, shoulder dystocia, meconium aspiration or cord prolapse; or abnormal fetal heart rate patterns during labour.

Exclusion criteria: intrauterine fetal death prior to hospital admission; termination of pregnancy for medical reasons; severe congenital anomalies that could potentially affect neonatal survival or long-term neurodevelopmental outcomes and neonatal encephalopathy not related to hypoxic-ischaemic origin.

### Primary outcome measure

The primary outcome will be the frequency of optimal versus suboptimal care observed during maternal and neonatal management in cases of moderate to severe HIE or neonatal death related to severe perinatal asphyxia.

Suboptimal care will be classified based on data entered in the electronic case report form (eCRF), using the framework described by Leape *et al*,^13^ encompassing the following categories:

- Diagnostic errors (errors or delays in diagnosis; failure to perform indicated diagnostic tests; use of outdated diagnostic methods; misinterpretation of diagnostic test results).
- Therapeutic errors (errors in procedural performance; errors in medication administration; dosage errors; avoidable delays in treatment or in responding to abnormal test results; provision of inappropriate care).
- Preventive failures (lack of indicated prophylactic treatment; inadequate monitoring or follow-up of treatment).
- Other systemic or organisational failures (communication failures; equipment deficiencies; other system-level failures).

### Secondary outcomes

#### Neonatal outcome

Neonatal outcome will be defined using a composite endpoint that includes both mortality (early neonatal death within the first 24 hours of life or during the initial hospitalisation prior to any potential discharge home) and/or severe neonatal morbidity (defined as the presence of brain lesions on MRI attributable to perinatal asphyxia according to the NICHD classification)^14^ and/or an abnormal electroencephalogram performed within the first week of life according to the classification of Lamblin *et al*^15^ and/or clinical abnormalities assessed using the adapted Sarnat classification at birth^16^ and subsequently using the Amiel-Tison neurological assessment during the first week of life.^17^

#### Characteristics of suboptimal care identified by expert reviewers

Suboptimal care will be analysed using the medical error classification proposed by Leape *et al*^18^ (as described in the primary outcome) with attention to characteristics indicative of systemic errors.

#### Determinants of suboptimal care

Determinants will be explored using a root cause analysis (RCA) approach. RCA focuses on identifying the initiating factor(s) that triggered the chain of events leading to patient harm. The aim is to highlight systemic factors contributing to the error,^10^ such as level of maternity unit, timing of care (eg, during night shifts), staff qualifications, equipment used, etc.

#### Assessment of the potentially avoidable nature of the case

Suboptimal care with a potentially avoidable complication refers to an event that could have been avoided through appropriate error management strategies.^19^ The assessment of avoidability includes a subjective component and will be determined by expert consensus based on the optimality of care provided. In the absence of consensus, inter-rater agreement will be measured using Cohen’s Kappa coefficient, with values >0.61 considered sufficient for this study. Cases will be classified into three categories: probably avoidable, probably unavoidable or undetermined.

#### Concordance between data collected in the eCRF and local MMR review versus confidential enquiry findings

This analysis will be both qualitative and quantitative, comparing the data reported in the eCRF completed by the referring clinician and the local MMR with the findings from the national-level confidential enquiry.

### Study organisation

**Figure 1:**
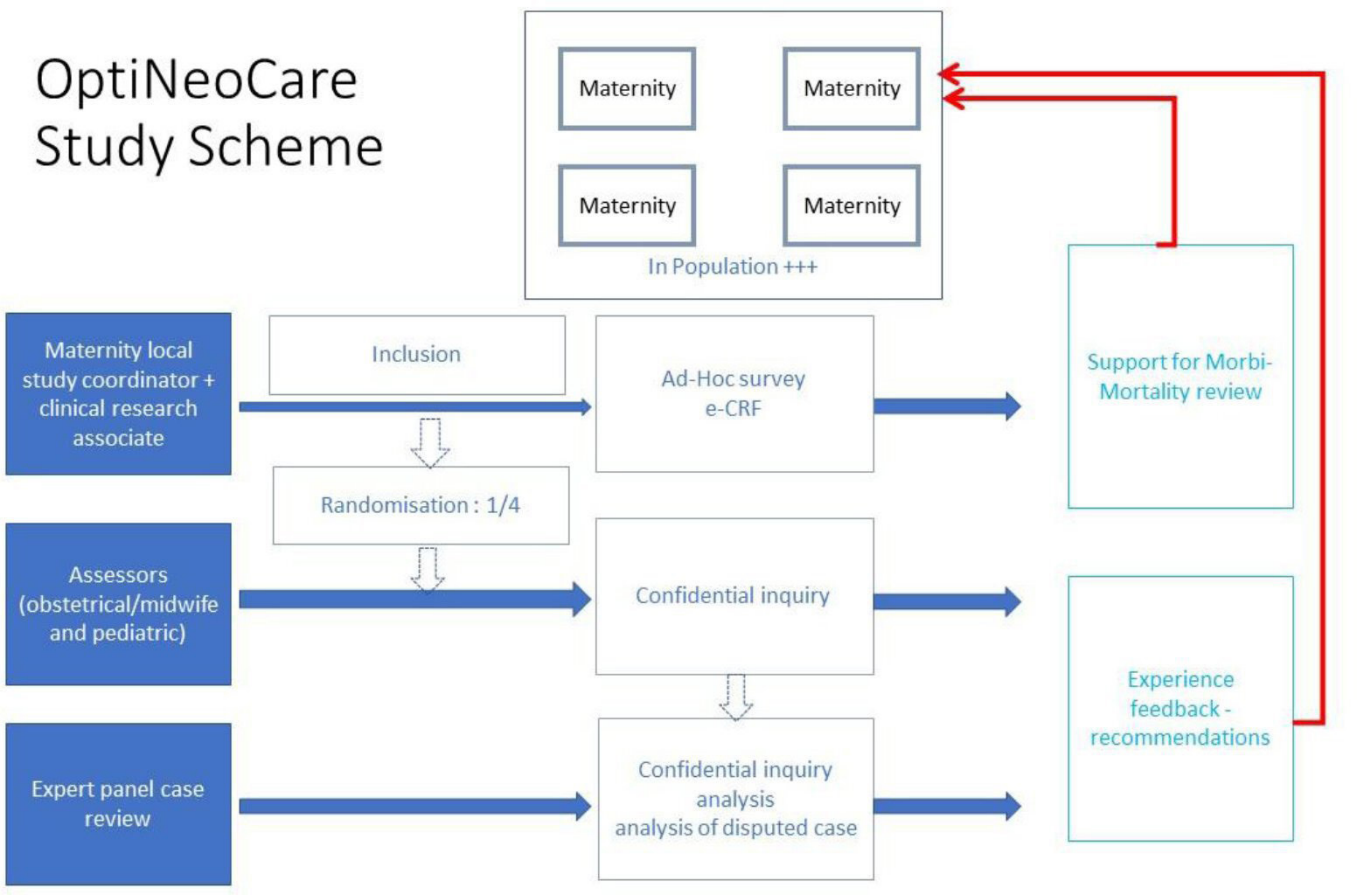
Schematic overview of the OptiNeoCare study. eCRF, electronic case report form.

All parents will be informed of the study through public notices (posters displayed in maternity wards and neonatal units) and via an individual information leaflet provided at admission. The designated clinician (obstetrician, neonatologist or midwife) will record in the medical file the absence of opposition from the mother to the collection of her personal data, as well as the absence of opposition from both parents regarding the collection of data concerning their newborn (figure 1).

In all participating centres, in the event of severe perinatal asphyxia, prospective data collection will be initiated following an interview between the family and the case’s referring physician or local study coordinator. During this interview, study information will be provided, and non-opposition to participation will be documented. Data collection will be carried out by the local study investigator at the maternity unit—potentially assisted by a clinical research technician (CRT) as early as possible after the event, no later than within the first week.

This prospective data collection will include:

- Maternity unit: labour and delivery room
  - Maternal and obstetric data: maternal medical history, prenatal care and risk factors associated with perinatal asphyxia.
  - Intrapartum obstetric and anaesthetic data: partogram, fetal heart rate monitoring and timestamped records of decisions and clinical interventions.
  - Neonatal data: initial clinical examination, detailed and timestamped description of delivery room management and administered treatments.
  - Organisational data: personnel present, structure and workflow, workload, available and completed documentation.
- Neonatal transport
  - Timestamped information on management, interventions and neonatal parameters during transport.
  - Organisational data: personnel involved, travel distance and duration.
- Neonatal intensive care
  - Timestamped data on management, treatments and neonatal status on arrival and during the first 24 hours of life.
  - Organisational data: personnel present and workload.

Following the inclusion of cases, a random selection of medical records will be subject to expert review. One in four cases will be randomly selected using the CleanWeb software. These ‘expert review’ cases will undergo a confidential enquiry by two assessors (an obstetrician and a neonatologist) who will visit the participating centres. Data will be collected from the medical records, available institutional documents and interviews with the healthcare professionals involved in the case. All collected information will be entered into the CleanWeb eCRF.

The data will be submitted to the OptiNeoCare study steering committee, which will verify data anonymisation before transmitting them in a digital format to two expert reviewers. These reviewers will be responsible for preparing the expert panel meeting related to the case. If needed, the reviewers may request additional information from the study steering committee, which will then forward the clarifications to the assessors.

During the expert panel meeting, the case files will be made available to the experts in both paper and electronic formats. The discussions will be summarised in a report, which will be electronically validated by the experts and sent every 4 months to the participating centres via the internet. This report will be accessible through a dedicated website for the study.

The MMR reports recorded in the e-CRF will be reviewed by the expert panel after the end of the inclusion period. The panel will summarise the main qualitative and quantitative findings in a comprehensive anonymised report.

### Statistical methods

#### Descriptive analysis

Quantitative variables will be described using counts, means, SD, medians, quartiles, ranges and the number of missing values. Qualitative variables will be presented as counts, percentages and the number of missing values.

The primary outcome (the frequency of optimal management) will be described and factors associated will be analysed using a logistic regression model. Secondary outcomes will be analysed using the same type of model as for the primary outcome. For continuous outcomes, mixed linear models will be used. An analysis of the determinants of suboptimal care will be performed using multivariable logistic regression to identify variables independently associated with suboptimal management, adjusting for relevant covariates. Given the method used to assess the primary outcome (expert classification of care quality), no missing data are expected for this variable. However, in rare cases where experts are unable to decide, a ‘cannot assess’ category will be available. Some missing data in the medical records will be considered meaningful and indicative of suboptimal care (eg, absence of documented clinical examination in the medical file). In such cases, the newborn will be classified as having received suboptimal care. For other variables, multiple imputation using chained equations may be applied to address missing data.

#### Sample size

The prevalence of HIE is estimated at 1–2 per 1000 live births.^20^ In the national French-based Lytonepal cohort, which included only cases of moderate to severe HIE, the prevalence was estimated at 0.90 per 1000 live births (95% CI: 0.84 to 0.95).^21^ For the OptiNeoCare study, we estimated a prevalence of 1 per 1000 births, as we also include intrapartum stillbirths and cases of HIE initially missed at birth but subsequently hospitalised for neurological symptoms. These cases were not captured in the Lytonepal cohort.

Furthermore, according to the literature, suboptimal care in the management of HIE is reported in approximately 50% of cases.^7 11 22^ We therefore hypothesise that about 50% of the care provided in cases of severe perinatal asphyxia is suboptimal. The study will include 12 perinatal centres covering a population with approximately 340 000 births per year. Level III maternity units and their associated centres are expected to include, on average, 1.5 newborns per month, corresponding to a total of approximately 336 infants over a 12-month inclusion period.

This recruitment will allow us to conduct a confidential enquiry on 84 infants, enabling the identification of suboptimal care in approximately 42 cases. In addition, the in-situ component of the study will provide data on the remaining 252 cases, allowing us to identify and describe around 126 instances of suboptimal care.

### Data quality control

Several procedures will be implemented to ensure data quality throughout the study. Standardised data collection forms and a centralised (eCRF) system (CleanWeb) will be used to minimise variability and entry errors. Data will be entered by trained local investigators or CRTs at each site. Automated consistency and plausibility checks will be integrated into the eCRF to identify outliers, inconsistencies or missing values in real time. Regular data monitoring will be conducted by the coordinating centre to verify the completeness, accuracy and timeliness of data entry. Queries will be generated and resolved in collaboration with the local teams.

Additionally, expert case reviews will include cross-checking of clinical documentation, organisational records and interviews with care teams, ensuring a comprehensive and high-fidelity dataset. Finally, participating centres will be regularly contacted by the national coordinating team via email to report any occurrences of severe perinatal asphyxia. This will include both maternity units and the associated neonatal intensive care units (NICUs) and transport teams. The collected data (place and date of birth) will be cross-checked in the CleanWeb system to prevent duplicate case inclusion.

At the end of the study, a query will be submitted to the Centre d’épidémiologie sur les causes médicales de décès (*CepiDC*), a comprehensive national mortality database, to verify that all cases of neonatal death prior to transfer have been appropriately identified and reported.

## Ethics and dissemination

Our protocol has received approval from the Ethics Committee for Research in Obstetrics and Gynaecology (CEROG): CEROG 2023-OBS-1206

Hospitals have signed multicentre collaboration agreements. Participants (or their legal representatives) will be thoroughly informed about this study, receiving written information and having the option to oppose the collection of data related to their case. In accordance with Article L1121-1-1 of the French Public Health Code, no non-interventional research may be conducted on an individual who has objected to participation after having received the information required under Article L1122-1 of the same code. The guardians of the newborns and the public will not be involved in the study design. However, newborns will be recruited voluntarily during the enrolment phase. Participation will be entirely voluntary. The study results will be disseminated to guardians and the public through public health education initiatives, as well as neonatal brain injury conferences and academic journals.

This research falls within the scope of the ‘Reference Methodology for the Processing of Personal Data in Health Research’ (modified MR-004). The study sponsor, Assistance Publique–Hôpitaux de Paris, has signed a compliance commitment to this reference methodology.

All documents and data collected during the research will be securely stored and retained for up to 2 years following the final publication of the study results. In the absence of publication, data will be kept until the final research report is signed. Data management procedures follow established standards to ensure confidentiality, integrity and traceability. Only authorised members of the research team will have access to the data. No personal identifying information will be published.

This study is registered on ClinicalTrials.gov under the ID: NCT06322732.

## Discussion

Severe perinatal asphyxia at term is a critical condition caused by obstetric events or fetal heart rhythm abnormalities during labour, that can result in intrauterine or peripartum death (0.6/1000 births) or HIE (1.6/1000 live births),^1^ affecting ~1000 newborns annually in France. As an unexpected and urgent situation, immediate obstetric and neonatal management is crucial for the prognosis of the newborn.^23^ Despite advances like therapeutic hypothermia in the neonatal critical care unit,^3^ the prognosis for HIE newborns remains poor, with a neonatal mortality risk estimated at 15–20% and a disability rate among survivors at 30%.^4^ This issue is particularly acute given that since 2012, France has seen an increase in the paediatric mortality rate,^24^ primarily related to perinatal deaths. Since 2015, infant mortality has been higher than the European average,^25^ with France, which ranked third among the European countries, now ranking 20th.^26^ This marker is recognised as one of the recommended indicators of perinatal health in European countries.^27^ Knowing the incidence of severe perinatal asphyxia and understanding its determinants are important public health challenges.

One of the strengths of this prospective observational study lies in its comprehensive approach, encompassing the entire continuum of obstetric and neonatal care, including neonatal transport. This design enables the identification of potential challenges in the coordination of care from a multiprofessional perspective.

Another key strength is the involvement of all maternity units within participating perinatal networks, including public and private hospitals of various levels. This is particularly relevant for a condition such as perinatal asphyxia, which can occur regardless of the type of facility where delivery takes place. Furthermore, this inclusive approach allows for the identification of potentially suboptimal care practices in centres that are traditionally underrepresented in clinical research in France.

Finally, the geographical diversity of participating regions—including urban areas, rural settings such as Burgundy and mountainous regions like the Grenoble network—will contribute to a comprehensive assessment of care quality across contrasting healthcare environments.

Among the potential challenges, ensuring the completeness of case inclusion and verifying this completeness is of particular importance. To minimise missed inclusions, inclusion monitoring will be carried out through the perinatal network. This will involve: (1) contacting participating maternity units to identify any potentially eligible but non-included cases and (2) querying the NICUs to which these centres refer, as well as the SMUR neonatal transport units, both of which are centrally coordinated within the French perinatal network. In addition, neonatal deaths are recorded in a national mortality database (CepiDC), which will be used to identify potential cases of death occurring prior to transfer.

Finally, this pioneering study on suboptimal care in the obstetric and neonatal management of severe perinatal asphyxia will provide critical insights into the circumstances and determinants of care failures. Beyond its immediate findings, it aims to establish a sustainable, nationwide system for systematic case analysis. This initiative is intended to drive continuous improvement in the quality of perinatal care and ultimately reduce the burden of morbidity and mortality associated with this serious condition.
