# Supplementary material for: OptiNeoCare: optimisation of routine care in the management of severe perinatal asphyxia in full-term or near-term newborns – study protocol for analysis of suboptimal care by confidential inquiries and e-self report
Source: BMJ Open. 2025 Jul 5;15(7):e106093. doi: 10.1136/bmjopen-2025-106093 (PMC12228471; doi:10.1136/bmjopen-2025-106093)
Supplement: Supplementary file 1 [file bmjopen-2025-106093.reviewer_comments.pdf]

PEER REVIEW HISTORY

BMJ Open publishes all reviews undertaken for accepted manuscripts. Reviewers are asked to complete a checklist review form and are provided with free text boxes to elaborate on their assessment. These free text comments are reproduced below.

ARTICLE DETAILS

Title (Provisional)

OptiNeoCare: Optimization of routine care in the management of severe perinatal asphyxia in full-term or near-term newborns: study protocol for analysis of suboptimal care by confidential inquiries and e-self report

Authors

Guellec, Isabelle; Ancel, Pierre-Yves; Abdoul, Hendy; Garabedian, Charles; Verspyck, Eric; Huissoud , Cyril; Delnaud, Marie; Graesslin, Blanche; Desplanches, Thomas; Cambonie, Gilles; Tourneux, P.; Debillon, Thieny; Mitha, Ayoub; Loron, Gauthier; Favrais, Géraldine; Badr, Maliha; Chapeliere, Sophie; Brasseur-Daudruy, Marie; Gonzalez Estevez, Max; Rigouzzo, Agnes; Delorme, Pierre; Kayem, Gilles

---

This article was not externally reviewed at BMJ Open. Protocols that have been independently assessed prior to submission to BMJ Open are usually fast-tracked to publication on the grounds that further substantial changes will not be possible. This independent assessment will usually be external, independent review for both a substantial grant award from a non-commercial or government funder and ethics approval.
